# Outcomes of neonatal congenital diaphragmatic hernia in a non-ECMO center in a middle-income country: a retrospective cohort study

**DOI:** 10.1186/s12887-022-03453-5

**Published:** 2022-07-07

**Authors:** Lucy Chai See Lum, Tindivanum Muthurangam Ramanujam, Yee Ian Yik, Mei Ling Lee, Soo Lin Chuah, Emer Breen, Anis Siham Zainal-Abidin, Srihari Singaravel, Conjeevaram Rajendrarao Thambidorai, Jessie Anne de Bruyne, Anna Marie Nathan, Surendran Thavagnanam, Kah Peng Eg, Lucy Chan, Mohamed E. Abdel-Latif, Chin Seng Gan

**Affiliations:** 1 Department of Pediatrics, University Malaya Medical Center, 59100 Lembah Pantai, Kuala Lumpur, Malaysia; 2 Division of Pediatric Surgery, Department of Surgery, University Malaya Medical Center, Kuala Lumpur, Malaysia; 3 Department of Pediatrics, Hospital Tengku Ampuan Afzan, Pahang, Malaysia; 4 Clinical Investigation Center, University of Malaya Medical Center, 5th Floor East Tower, Kuala Lumpur, Malaysia; 5 Department of Pediatrics, Universiti Teknologi MARA, Selangor, Malaysia; 6 Queen Mary University of London, Barts Health NHS Trust, Royal London Children’s Hospital, London, UK; 7 Department of Anesthesia, University Malaya Medical Center, Kuala Lumpur, Malaysia; 8 Department of Neonatology, Centenary Hospital for Women and Children, The Canberra Hospital, Canberra, ACT Australia; 9 Department of Public Health, La Trobe University, Bundoora, Melbourne, VIC Australia

**Keywords:** Hernias, Diaphragmatic, Congenital, Infant, Newborn, Intensive care units, Pediatric, Prenatal diagnosis, Risk factors, Survival

## Abstract

**Background:**

Most studies examining survival of neonates with congenital diaphragmatic hernia (CDH) are in high-income countries. We aimed to describe the management, survival to hospital discharge rate, and factors associated with survival of neonates with unilateral CDH in a middle-income country.

**Methods:**

We retrospectively reviewed the medical notes of neonates with unilateral CDH admitted to a pediatric intensive care unit (PICU) in a tertiary referral center over a 15-year period, from 2003–2017. We described the newborns’ respiratory care pathways and then compared baseline demographic, hemodynamic, and respiratory indicators between survivors and non-survivors. The primary outcome measure was survival to hospital discharge.

**Results:**

Altogether, 120 neonates were included with 43.3% (52/120) diagnosed antenatally. Stabilization occurred in 38.3% (46/120) with conventional ventilation, 13.3% (16/120) with high-frequency intermittent positive-pressure ventilation, and 22.5% (27/120) with high frequency oscillatory ventilation. Surgical repair was possible in 75.0% (90/120). The overall 30-day survival was 70.8% (85/120) and survival to hospital discharge was 66.7% (80/120). Survival to hospital discharge tended to improve over time (*p* > 0.05), from 56.0% to 69.5% before and after, respectively, a service reorganization. For those neonates who could be stabilized and operated on, 90.9% (80/88) survived to hospital discharge. The commonest post-operative complication was infection, occurring in 43.3%. The median survivor length of stay was 32.5 (interquartile range 18.8–58.0) days. Multiple logistic regression modelling showed vaginal delivery (odds ratio [OR] = 4.8; 95% confidence interval [CI] [1.1–21.67]; *p* = 0.041), Apgar score $\ge$ 7 at 5 min (OR = 6.7; 95% CI [1.2–36.3]; *p* = 0.028), and fraction of inspired oxygen (FiO_2_) < 50% at 24 h (OR = 89.6; 95% CI [10.6–758.6]; *p* < 0.001) were significantly associated with improved survival to hospital discharge.

**Conclusions:**

We report a survival to hospital discharge rate of 66.7%. Survival tended to improve over time, reflecting a greater critical volume of cases and multi-disciplinary care with early involvement of the respiratory team resulting in improved transitioning from PICU. Vaginal delivery, Apgar score $\ge$ 7 at 5 min, and FiO_2_ < 50% at 24 h increased the likelihood of survival to hospital discharge.

## Background

Congenital diaphragmatic hernia (CDH) is a severe diaphragmatic malformation that permits abdominal organ herniation into the chest cavity. It is rare, with population studies giving an estimated prevalence of 2.3 per 10,000 live births [1], and life-threatening, with varying outcomes depending on a wide range of physiological severities.

Treatment of CDH is challenging, requiring the expertise of integrated multidisciplinary teams in well-resourced intensive care units. The aim is to gradually recruit the lungs and simultaneously minimize lung injury, which can be achieved using ‘gentle ventilation’ with permissive hypercapnia [2–4]. High-frequency oscillation ventilation (HFOV) decreases morbidity due to air leak and contralateral pneumothorax, which can further compromise the residual hypoplastic lung [2–4]. Pharmacological treatment of pulmonary hypertension using inhaled nitric oxide (iNO) and other intravenous vasodilators, such as milrinone, are widely used with varying hemodynamic responses. Cardio-respiratory stabilization before surgical repair, is a well-established practice associated with improved outcomes [4, 5]. Extracorporeal membrane oxygenation (ECMO) is used in some countries to support these neonates but is not typically available in lower- and middle-income countries. Indeed, the survival benefit of ECMO may be confined to more severe cases of CDH [6]. Antenatal interventions such as fetal endoscopic tracheal occlusion [7] and termination of pregnancy [3, 8, 9] are not available in our center.

Population-based studies from the 1970s to 1990s, which included elective termination and still-born neonates, consistently reported CDH mortality rates ranging from 61–66% [3, 9, 10]. Although more recent analyses suggest an improvement in survival, selection bias is likely. Indeed, a population-based study shows an inverse correlation between the rate of antenatal deaths, principally elective termination, and the overall mortality rate of CDH live births [9]. The presence of co-existing major anomalies can reduce survival to 22% [3].

The CDH Study Group (CDHSG) initiated a registry, collecting multi-institutional data from many countries, to evaluate variations in treatment and outcome, with the ability to adjust for severity [11]. The registry data over time show a decreasing use of ECMO and an increasing use of iNO. From 2007 to 2019, the overall survival of CDH liveborn neonates was 72% [12]. However, patients continue to spend considerable time in hospital and suffer from significant morbidity and mortality, particularly those with severe defects [13].

Over the past two decades, studies from South and Southeast (SE) Asian countries, mostly involving relatively small numbers of neonates and with varying selection criteria, report survival rates ranging from 56–78% in India [14–18], 52% in Malaysia [19], and 56–79% in Taiwan [20, 21] and Singapore [8, 22], moving from low-middle, upper-middle, and high-income countries, respectively. Antenatal diagnosis varies between 5–79% in these studies and risk factors for non-survival include an antenatal diagnosis, low Apgar score, the presence of moderate-to-severe persistent pulmonary hypertension of the newborn (PPHN), pneumothorax, and high oxygenation index (OI). Many of these identified risk factors are univariate associations.

The objectives of this study were to describe the management and survival rates of neonates with CDH admitted to a non-ECMO pediatric intensive care unit (PICU) in a middle-income country in SE Asia over a 15-year period, and to analyze the factors associated with survival.

## Methods

We performed a retrospective cohort study including all liveborn neonates diagnosed with unilateral CDH from 2003–2017, admitted to the PICU, University Malaya Medical Center, Kuala Lumpur, Malaysia.

The respiratory care strategy for newborns diagnosed with CDH followed a standard protocol, which was modified over the 15-year period as new evidence and infant ventilators became available. All infants were intubated as soon as the CDH diagnosis was recognized and ventilated with conventional ventilation using a SERVO*-*i ventilator (MAQUET Critical Care AB, Solna, Sweden). From 2015, a Fabian Ventilator (Acutronic Medical Systems AG, Hirzel, Switzerland) was added. The ventilation strategy followed an established protocol based on Wung et al. [5] and Chu et al. [21]: respiratory rates of 40 to 60 breaths/min, positive end-expiratory pressure (PEEP) of 5 cm H_2_O, and pressure above PEEP of 15 to 20 cm, to obtain a peak inspiratory pressure (PIP) of 20 to 25 cm H_2_O for adequate chest/abdominal excursion. Supplemental oxygen was administered to maintain pre-ductal oxygen saturation (SpO_2_) > 90%. Permissive hypercapnia with a pH > 7.25 was considered acceptable. Neonates who did not respond were switched to high-frequency intermittent positive-pressure ventilation (HIPPV) using the same ventilator but with a respiratory rate of 100 breaths/min, pressure above PEEP of up to 20 cm H_2_O, and PEEP of 0 cm H_2_O [5, 21] but with a targeted auto-PEEP of 5 cm H_2_O. In cases with persistent hypoxemia, labile oxygenation, or if the partial pressure of carbon dioxide (PaCO_2_) > 60 mm Hg, HFOV was used as the rescue mode [21].

In the presence of PPHN, iNO therapy at 20 ppm was initiated [23, 24]. Magnesium sulphate infusion was added when the response to iNO therapy was suboptimal [25]. Patients were sedated with morphine and midazolam infusions with intermittent doses of ketamine as needed. Muscle relaxants were avoided in most patients and given in intermittent doses if required for patient-ventilator synchrony. The goal of the strategy was to achieve a pre-ductal SpO_2_ > 88% with minimal but adequate ventilator settings. Hyperventilation to induce alkalosis and hyperinflation of the hypoplastic lungs were avoided by reducing the ventilation rate and the pressure above PEEP. Inotropic support using dopamine, noradrenaline, adrenaline, and/or milrinone, and calcium infusions were used to maintain mean arterial blood pressure ≥ 40 cm H_2_O, which minimized right-to-left shunting across the ductus arteriosus. Echocardiography to monitor heart function, intravascular volume status, right and left ventricular functions and to diagnose coexisting cardiac defects was conducted as soon as the neonate’s condition permitted.

Surgery was performed when the neonate’s general condition and hemodynamic parameters stabilized for at least 24 h. After transitioning to conventional ventilation and a fraction of inspired oxygen (FiO_2_) < 50%, the patient was weaned off iNO and all inotropes. Post-operatively, patients were continued on conventional ventilation and when stabilized were extubated to non-invasive ventilation.

In 2007, a service reorganization occurred resulting in changes to the management of a pregnancy complicated with CDH and care of the neonate. This included the establishment of a dedicated antenatal counselling service, advanced preparation by the PICU team when the mother was in early labor, and early involvement of the respiratory team who managed the infant’s post-PICU care.

### Variable selection

All neonates with CDH were identified from PICU admission records, their case notes reviewed, and the following information was extracted: baseline demographic and perinatal characteristics (see Table 1), and pre-operative respiratory and hemodynamic indicators (see Table 2) including: type of ventilation method chosen (conventional ventilation, HIPPV, HFOV), response to ventilation (a positive response was defined as maintaining preductal SpO_2_ > 88 mm Hg**),** surgical repair (yes/no), stabilization achieved prior to surgery (yes/no), defect size (A, B, C, D) as used by the CDHSG [26], type of repair (primary/synthetic patch or muscle), presence of PPHN as diagnosed clinically and confirmed by Doppler echocardiography (yes/no), the presence of a co-existing significant abnormality (i.e., a major cardiac or syndromic anomaly), and the presence and type of post-op complications. The main outcome was survival to hospital discharge. Secondary outcomes were 30-day survival, length of hospital stay, and risk factors associated with survival.

**Table 1:** Demographic and perinatal characteristics of patients with congenital diaphragmatic hernia

Characteristics	*n* = 120 (%)
**Male**	71 (59.2)
**Female**	49 (40.8)
**Ethnicity**	
**Malay**	53 (44.2)
**Chinese**	31 (25.8)
**Indian**	32 (26.7)
**Others**	4 (3.3)
**Left-sided hernia**	112 (93.3)
**Time of Diagnosis**	
**Prenatal Diagnosis**	52 (43.3)
**Postnatal Diagnosis**	68 (56.7)
**Place of delivery**	
**Inborn**	59 (49.2)
**Outborn**	61 (50.8)
**Gestational age, median (IQR), weeks**^**a**^	38.1 (37–40)
**Term (≥ **$37$**weeks)**	105 (89)
**Preterm (< 37 weeks)**	13 (11)
**Mode of delivery**	
**Vaginal**	73 (60.8)
**Emergency Cesarean section**	33 (27.5)
**Elective Cesarean section**	14 (11.7)
**Birth weight, median (IQR), g**^**b**^	2940 (2585–3200)
**Apgar score ≥ 7 at 1 min**^**c**^	59 (51.3)
**Apgar score ≥ 7 at 5 min**^**c**^	86 (74.8)
**Associated significant anomalies**^**d**^	
**Cardiac**	7 (5.9)
**Syndromic**	12 (10.1)
**Time of death, median (IQR), days (*n* = 40)**	2 (1–11)
**Surgical repair (*n* = 90)**	
**Hernia type**	
Bochdalek	69 (76.7)
Central	5 (5.6)
Other	16 (17.8)
**Defect size**^**a**^**:**	
**A**	7 (8.0)
**B**	66 (75.0)
**C**	15 (17.0)
**D**	0 (0.0)
**Repair**	
**Primary**	71 (78.9)
**Synthetic patch**	13 (14.4)
**Muscle flap**	6 (6.7)
**Age of repair, median (IQR), days**^**d**^** (*n* = 90)**	7 (5–12)
**Survivor length of stay, median (IQR)**^**a**^**, days (*n* = 80)**	32.5 (18.8–58.0)

Number of cases with missing data: ^a^2, ^b^4, ^c^5, ^d^1 IQR, interquartile range

**Table 2:** Preoperative comparisons between survivors and non-survivors to hospital discharge

Categorical variables	Survival to hospital discharge		*p*-value
	**Yes n (%)**	**No, n (%)**	
**Prenatal diagnosis (*n* = 120)**	26 (32.5)	26 (65.0)	**0.001**
**Inborn (*n* = 120)**	31 (38.8)	28 (70.0)	**0.001**
**Birth lower segment Cesarean section (*n* = 120)**	25 (31.3)	22 (55.0)	**0.012**
**Apgar score at 1 min < 7 (*n* = 115)**	28 (36.8)	28 (71.8)	** < 0.001**
**Apgar score at 5 min < 7 (*n* = 115)**	12 (15.8)	17 (43.6)	**0.001**
**Response to conventional ventilation (*n* = 120)**	44 (55.0)	2 (5.0)	** < 0.001**
**Response to HIPPV (*n* = 49)**	14 (60.9)	2 (7.7)	** < 0.001**
**Response to HFOV (*n* = 50)**	22 (100)	5 (17.9)	** < 0.001**
**Pulmonary hypertension (*n* = 119)**	50 (62.5)	36 (92.3)	**0.001**
**Pre-operative air leak (*n* = 114)**	7 (9.1)	10 (27.0)	**0.012**
**At surgery (*n* = 90)**			
**Low-risk defect (A or B) (*n* = 88)**	69 (88.5)	4 (40.0)	**0.001**
**Primary repair (*n* = 90)**	68 (85.0)	3(30.0)	**0.001**
**Significant anomaly** (*n* = 119)	9 (11.3)	9 (23.1)	0.091
FiO_2_ at 24 h < 50% (*n* = 98)	54 (77.1)	1(3.6)	** < 0.001**
**Continuous variables**	**median (IQR)**	**median (IQR)**	
**Birth weight, g (*n* = 116)**	2978 (2650–3260)	2900 (2500–3000)	0.692
**Gestational age, weeks (*n* = 118)**	38.3 (37.0–40.0)	38.0 (37.0–40.0)	0.845
**Pre-ductal SpO**_**2**_** at 24 h, % (*n* = 36)**	99.0 (98.0–100.0)	94.0 (90.5–98.5)	**0.014**
**Post-ductal SpO**_**2**_** at 24 h, % (*n* = 37)**	88.0 (68.0–95.0)	52.0 (91.0–98.0)	** < 0.001**
**Initial pH (*n* = 112)**	7.3 (7.2–7.5)	7.4 (6.9–7.2)	** < 0.001**
**Initial pCO**_**2**_**, mmHg (*n* = 112)**	53.5 (40.9–70.7)	74.4 (54.5–112.5)	**0.004**
**Initial pO**_**2**_**, mm Hg (*n* = 111)**	52.1 (41.7–68.6)	39.1 (28.2–59.3)	**0.005**

*FiO*_*2*_ Fraction of inspired oxygen, *HIPPV* High-frequency intermittent positive-pressure ventilation, *HFOV* High-frequency oscillation ventilation, *IQR* Interquartile range, *pCO*_*2*_ Partial pressure of carbon dioxide, *pO*_*2*_ Partial pressure of oxygen, *SpO*_*2*_ Oxygen saturation, *SD* Standard deviation

### Statistical analysis

A descriptive analysis was undertaken for all study participants. Categorical data are described using numbers and percentages. The Shapiro–Wilk test was used to assess normal distribution. If continuous data were normally distributed, they are described using mean and standard deviation (SD); if not normally distributed, median, and interquartile range (IQR) are used. Univariate associations between pre-operative respiratory, pre-operative hemodynamic, and surgical characteristics and survival to hospital discharge were assessed using the chi-squared test for categorical data (Fisher’s exact test if *n* < 5). For continuous data, the Mann–Whitney U test was used. Univariate associations with survival were assessed. Adjusted differences in survival, based on pre-operative indicators, were estimated utilizing multiple logistic regression models. Criteria for entry and removal from this model were a *p* < 0.05 and *p* > 0.10, respectively. The final multivariate model chosen was one that best represented the data. Data were analyzed using SPSS Statistics for Windows, version 23.0 (IBM Corp., Armonk, NY, USA). *P*-values < 0.05 were considered statistically significant.

## Results

Altogether 120 neonates were diagnosed with CDH, with 59.2% males (71/120). See Table 1. Based on chest radiography, 93.3% (112/120) defects occurred on the left side. Among the 90 patients who underwent surgical repair, the commonest type of CDH was a Bochdalek hernia in 76.7% (69/90), followed by other herniation in 17.8% (16/90), and central herniation in 5.6% (5/90). A prenatal diagnosis of CDH was made in 43.3% (52/120) of neonates, while inborn cases constituted 49.2% (59/120). An antenatal diagnosis was significantly more likely to have occurred in inborn rather than outborn neonates, present in 98.1% (51/52) and 1.9% (1/52), respectively (*p* < 0.001). None of the children who were referred to us antenatally proceeded with elective termination. Altogether 41.2% of neonates (48/117) had associated anomalies, but only 14.5% (17/117) were deemed significant cardiac or syndromic anomalies — as defined by the CDHSG [11] — in 5.1% (6/117) and 9.4% (11/117) cases, respectively.

### Respiratory care

**Fig. 1:**
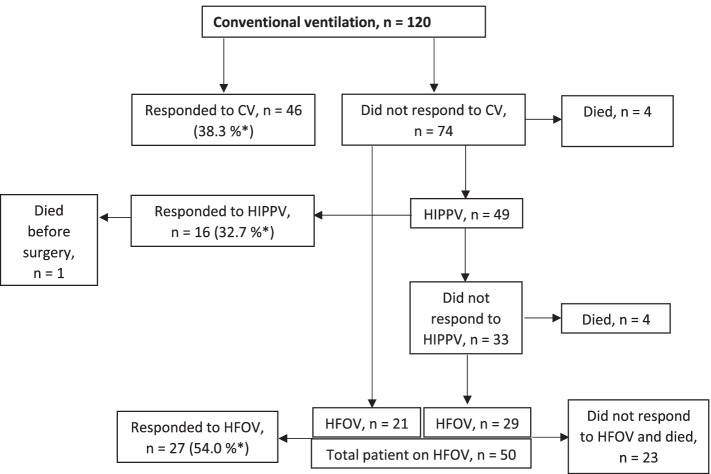
Respiratory Care Strategy Flowchart for Neonates with Congenital Diaphragmatic Hernia. * = Percentage of the total number of neonates for this ventilation mode. Abbreviations: CV, conventional ventilation; HIPPV, high-frequency intermittent positive-positive ventilation; HFOV, high-frequency oscillation ventilation

Figure 1 shows the flow of respiratory care therapy. Stabilization, with conventional ventilation at rates up to 60 breaths/min, was achieved in 38.3% (46/120), with HIPPV in 13.3% (16/120), and with HFOV in 22.5% (27/120), constituting a total of 74.2% (89/120) neonates. Of the remaining (31/120) neonates who did not achieve stabilization, 93.5% (29/31) were not operated on and died. The remaining two neonates underwent surgery despite not being stable; however, they both died postoperatively. One child was stabilized with HIPPV but died of sepsis before surgery.

### Survival

Surgical repair was carried out in 75.0% (90/120) patients, including two cases who did not achieve stabilization pre-operatively, at a median age of 7.0 (IQR 5.0–12.0) days. The remaining 25.0% (30/120) of cases died without operation. Of those who underwent surgical repair (*n* = 90), five neonates died before 30 days-of-life (including the two that were not stabilized pre-operatively; all died of PPHN, one had additional bowel ischemia, another had associated sepsis). Of the five patients who died after 30 days, two died without leaving PICU, one of PPHN and one of tracheobronchomalacia; the remaining three died after discharge from PICU, one of aspiration pneumonia due to a dislodged feeding tube, one of tracheobronchomalacia, and one of complications post-surgery for an interrupted aortic arch. In total, 69.2% (83/120) were discharged alive from PICU.

The overall 30-day survival was 70.8% (85/120), and the survival to hospital discharge rate was 66.7% (80/120). Of all those neonates who were stabilized and operated on, 90.9% (80/88) survived until hospital discharge. For those discharged home, the median length of hospital stay was 32.5 (IQR 18.5–58.0) days.

### Post-op complications

Post-operative complications were present in 47.8% (43/90) neonates. The commonest complication was blood stream infection 20.0% (18/90), followed by ventilator associated pneumonia 12.2% (11/90), wound or surgical site infection 10% (9/90), pneumothorax 3.3% (3/90), small bowel ischemia 1.1% (1/90), and infection site unspecified 1.1% (1/90). Those who survived to hospital discharge tended to have less complications than those who did not (45% vs. 70%, respectively; *p* = 0.184). Those with no complications had a significantly lower median length of hospital stay than those who had complications (26 days vs. 54 days; *p* < 0.001). Four infants required additional surgery within this admission: two required aortopexy for tracheobronchomalacia, one had repair of an interrupted aortic arch, and one had ligation of a large patent ductus arteriosus.

### The impact of service reorganization

The outcomes were analyzed into two time periods: 2000–2007 (period 1) and 2007–2017 (period 2), before and after a service reorganization, respectively. The number of cases antenatally diagnosed was significantly higher in period 2 compared with period 1 at 48.4% (46/95) vs. 24% (6/25), respectively, *p* = 0.028. Survival to discharge tended to improve with time at 69.5% (66/95) in period 2 from 56.0% (14/25) in period 1 (*p* > 0.05). The percentage of low-risk defects (size A/B) was similar across both time periods, 81.2% (13/16) and 83.3% (60/72) for periods 2 and 1, respectively, (*p* > 0.05) Furthermore, there were significant reductions in median length of hospital and PICU stays in period 2 compared with period 1. The median length of hospital PICU stay was 21 (IQR 11–36.3) days vs. 33 (IQR 20.5–53.3) days (*p* = 0.044) and the median length of hospital stay was 31 (IQR, 18–52.0) vs. 72 (IQR 39.5–133.5) days (*p* = 0.005), for period 2 compared with period 1, respectively.

### Univariate and multivariate analysis

Table 2 shows the differences in variables between survivors and non-survivors to hospital discharge. The presence of a congenital abnormality was not associated with survival (*p* > 0.05). Factors significantly associated with non-survival were antenatal diagnosis, being inborn, 1- and 5-min Apgar scores < 7, Cesarean section (elective and emergency) as the mode of delivery, high-risk defect (C or D), presence of pulmonary hypertension or preoperative air leak, non-stabilization preoperatively, lower initial pO_2_, lower initial pH, higher initial pCO_2_, and at 24 h lower pre- and post-ductal SpO_2_ and FiO_2_ $>$ 50%. For those neonates operated on, low-risk defect and primary repair were significantly associated with improved survival on univariate analysis (both *p* = 0.001).

**Fig. 2:**
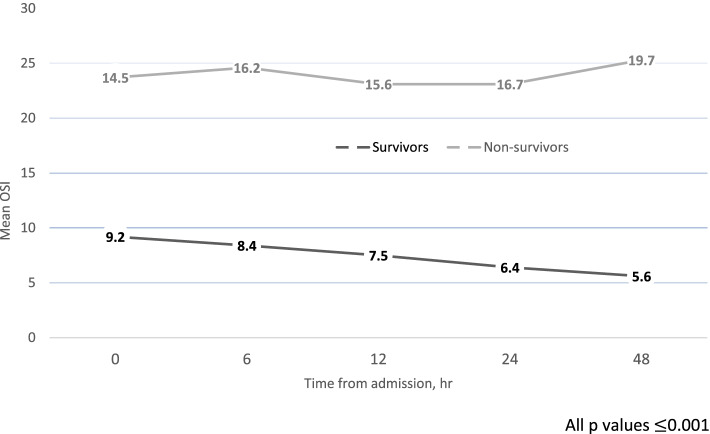
Comparison of Oxygen Saturation Index Between Survivors and Non-Survivors in First 48 h of Admission. For each time interval the mean OSI is significantly higher among non-survivors than survivors, *p* ≤ 0.001. Abbreviation: OSI, oxygen saturation index

Figure 2 shows the relationship between the preductal oxygen saturation index (OSI) and survival to hospital discharge. For the 0-, 6-, 12-, 24-, and 48-h intervals after admission, survivors had a significantly lower OSI compared with non-survivors (all *p* = 0.001 or less). A similar pattern was shown for OI; however, values were only significant at 0 and 6 h (both *p* < 0.001).

The variables used to predict survival in the final logistic regression model are shown in Table 3. Vaginal delivery (odds ratio [OR] = 4.8; 95% confidence interval [CI] [1.1–21.67]; *p* = 0.041), Apgar score $\ge$ 7 at 5 min (OR = 6.7; 95% CI [1.2–36.3]; *p* = 0.028), and FiO_2_ < 50% at 24 h (OR = 89.6; 95% CI [10.6–758.6]; *p* < 0.001) were significantly associated with survival to hospital discharge.

**Table 3:** Variables predicting survival in the multivariate logistic regression model

Variable	Odds Ratio	Confidence interval	*p*-value
**Vaginal delivery**	4.8	1.1–21.6	**0.041**
Apgar score at 5 min $\ge$ **7**	6.7	1.2–36.3	**0.028**
**FiO**_**2**_** < 50% at 24 h**	89.6	10.6–758.6	** < 0.001**

*FiO*_*2*_ Fraction of inspired oxygen

## Discussion

We described the management and survival of 120 liveborn neonates with unilateral CDH over a 15-year period, in our tertiary referral center PICU, in a middle-income country. Altogether, three-quarters of patients had surgical repair and two-thirds survived to hospital discharge. The median length of hospital stay for survivors was 32.5 days. Vaginal delivery, Apgar score $\ge$ 7 at 5 min, and FiO_2_ < 50% at 24 h were significantly associated with survival to hospital discharge.

The survival to hospital discharge rate in our center at 66.7% was lower than the 72% rate found in the CDHSG, the latter study having 6948 neonates spanning 12 years until mid- 2019; however, this difference was not statistically significant (*p* > 0.05) [12]. This registry incorporates the results of 90 centers in 18 mainly high-income countries, including some that have access to ECMO, not available in our country [11]. Altogether, the CDHSG treated 28% of neonates with ECMO and significantly more infants were surgically repaired, 84% vs. 75% in our study (*p* < 0.006) [12]. The prognosis for our patients who could be stabilized and operated on was excellent, with 90.9% surviving to hospital discharge. However, the CDHSG neonates who were operated on had a significantly lower percentage of neonates with defect size A/B compared with our neonates, 52.8% vs. 83.0%, respectively, *p* < 0.001 [12]. Low-risk defects are known to be associated with improved survival [26]. Conversely, larger defect sizes are associated with increased multi-system morbidity at discharge. The defect size in our study was evaluated retrospectively in most cases, based on descriptions and hand-drawings in post-operative notes, thus the defect sizes might not be accurate.

The survival to discharge rate tended to improve with time in our unit, reaching around 70% in the last ten years of analysis. This may be due to several factors following a service reorganization from 2007: the provision of a dedicated multi-disciplinary, antenatal counselling service; greater experience and organization of the team; improved transitioning from the higher-intensity PICU to the lower-intensity pediatric ward with early involvement of the respiratory team; and increasing recognition of our center as one that manages CDH, resulting in a greater patient volume over time. It has been recognized that a critical volume of at least six cases annually may partly explain better survival in some centers [27], particularly in cases diagnosed antenatally [28]. Our median survivor length of hospital stay following service reorganization (31 days) compares favorably with that for non-ECMO patients in high-income countries [29]. The survivors’ length of hospital and PICU stays were significantly reduced following our service reorganization, suggesting that the improved transition of care by the respiratory team on step down from PICU care contributed to an earlier hospital discharge. This ‘continuum of patient safety’ following PICU discharge has been identified as a factor that could reduce PICU readmission and mortality [30]. Other benefits of reduced hospital stay may include reductions in nosocomial infection and treatment cost as well as physical and psychological benefits for the family.

This is the largest study of CDH patients that we have found in SE Asia. Other smaller SE Asian studies have shown survival of patients ranging from 52–79% [8, 19–22]. Two of these non-ECMO centers in Taiwan and Singapore, which are high-income countries, report survival to hospital discharge rates of 79% [8, 20]. However, results from these studies could be biased by small numbers of liveborn neonates (*n* = 24 for both) and may reflect differing antenatal detection rates, termination rates, associated birth anomalies, ease of access to tertiary care facilities, and clinical management. Another study from Singapore found that 46% of cases diagnosed antenatally underwent termination [22]; this may in turn lead to improved survival rates of liveborn neonates with CDH. It is not surprising that of the 52 neonates diagnosed antenatally and referred to our hospital for counselling, none underwent termination. This reflects the cultural beliefs of our patient population, and that abortion is legal in Malaysia only under particular circumstances related to maternal health.

A recently published Danish study spanning a similar period, of equal size to ours, based in a tertiary referral unit without ECMO, and consisting of a similar percentage of antenatally detected cases had a 78% one-year survival [31]. Our study had a much higher rate of associated major congenital anomalies compared with the Danish study, 16.0% vs. 6%, respectively. Although we were unable to find that the presence of major anomalies was linked to reduced survival, the presence of associated cardiac and other malformations is linked to reduced survival elsewhere [32, 33]. Postoperative survival rates of stabilized neonates who had surgery in the Danish study were equivalent to our findings (89.2% vs. 90.9%, respectively).

We found that vaginal delivery was associated with a significantly increased chance of survival. In our center, CDH neonates with an antenatal diagnosis were permitted to deliver vaginally unless there were specific contraindications. Typically, the pregnant woman was induced at 38/39 weeks’ gestation, if spontaneous onset of labor had not yet occurred. Although the evidence supporting the best route is uncertain, a spontaneous vaginal delivery maximizes gestational maturity [34]. However, of those antenatally diagnosed in our study, only 51.9% had vaginal delivery compared with 67.6% of postnatally diagnosed neonates (*p* = 0.08). Indeed, in this antenatally diagnosed subgroup, survival remained higher following vaginal delivery at 65.4% (17/27) compared with Cesarean section at 34.6% (9/25), but the significance was borderline (*p* = 0.052). By contrast, antenatally diagnosed cases in the CDHSG showed a tendency for Cesarean section to improve survival, though findings were not significant; the authors excluded those with major heart defects and highlighted potential bias as centers that favor Cesarean section may have better survival rates [35]. However, in this latter study, Cesarean section delivered neonates were significantly more likely to survive without ECMO than those with vaginal delivery.

Ultimately neonatal survival will depend on the degree of hypoplasia of the lungs and the ability to recruit them with ventilation and without causing pulmonary injury. It is unsurprising that univariate risk factors for mortality include a lack of response to any of the graded ventilation strategies and indicators such as OSI at all time intervals up to 48 h after delivery. As an indicator of hypoxemic respiratory failure, OI has been shown to predict mortality in CDH [36]. We showed that OSI, a less invasive measurement, and a good predictor of OI [37], was significantly associated with mortality at a univariate level. Incomplete data may have contributed to a lack of significance at multivariate level.

An immediate postnatal marker of respiratory function, Apgar score $\ge$ 7 at 5 min, and early indicator, FiO_2_ < 50% at 24 h, were significantly associated with survival. These indicators have been found to significantly affect survival in studies of larger cohorts, are easily measured at the bedside, and are used in CDH survival prediction tools [38]. An FiO_2_ of < 50% at 24 h was chosen as a variable for analysis because it has been suggested that starting ventilation with this FiO_2_ value may be more beneficial than at 100% [39]. Of all the neonates that died, none were able to achieve an FiO_2_ < 50% in the first 24 h, while only 22.9% (16/70) of survivors had an FiO2 > 50% at this time.

Almost half of neonates who had surgery had a post-operative complication; 38.0% had infection. These are vulnerable neonates with immunological immaturity, undergoing multiple invasive procedures. A prospective observational study of CDH neonates in a neonatal intensive care unit in France (*n* = 62) had a 45% infection rate, with no associations with mortality but significant associations with prolonged stay [40]. Similarly, we found no associations with infection and survival to hospital discharge but found significant associations with length of hospital stay.

Limitations of this study are recognized. This is a single center, retrospective study, looking at live births that reached the PICU (excluding spontaneous abortion, stillbirth, or death before arrival). We have measured short-term outcomes only with no assessment of long-term morbidities, which can be considerable [8, 12]. Some of our data were incomplete and we failed to differentiate between induced and spontaneous vaginal delivery. Our database did not capture changes towards more minimally invasive surgery during the study period, which could have influenced survival and other outcomes. For post-operative complications, only the most significant infection was recorded; in reality, these neonates may have multiple infections. Future studies, ideally prospective ones, are needed to understand longer-term survival and assess morbidities associated with CDH repair. Our hospital has since joined the CDHSG, which standardizes the data captured, and this will help add to the global picture of CDH outcomes.

## Conclusions

In this middle-income country, neonates with unilateral CDH who reached PICU and were stabilized had an excellent chance of surviving to hospital discharge. Vaginal birth route, Apgar score $\ge$ 7 at 5 min, and FiO_2_ < 50% at 24 h were significantly associated with survival to hospital discharge.

## Data Availability

The dataset analyzed during the current study is available from the corresponding author on reasonable request.

## Abbreviations

CDH: Congenital diaphragmatic hernia
CDHSG: Congenital Diaphragmatic Hernia Study Group
CI: Confidence interval
ECMO: Extracorporeal membrane oxygenation
FiO_2_: Fraction of inspired oxygen
HFOV: High-frequency oscillation ventilation
HIPPV: High-frequency intermittent positive-pressure ventilation
iNO: Inhaled nitric oxide
OI: Oxygenation index
OSI: Oxygen saturation index
OR: Odds ratio
PaCO_2_: Partial pressure of carbon dioxide
pCO2: Partial pressure of carbon dioxide
PEEP: Positive end-expiratory pressure
PICU: Pediatric intensive care unit
PIP: Peak inspiratory pressure
pO2: Partial pressure of oxygen
PPHN: Pulmonary hypertension of the newborn
SD: Standard deviation
SE: Southeast
SpO_2_: Oxygen saturation
